# Iron Oxide Nanoparticles as Theranostic Agents in Cancer Immunotherapy

**DOI:** 10.3390/nano11081950

**Published:** 2021-07-29

**Authors:** Rossella Canese, Federica Vurro, Pasquina Marzola

**Affiliations:** 1 MRI Unit, Core Facilities, Istituto Superiore di Sanità, 00161 Rome, Italy; 2 Department of Computer Science, University of Verona, 37134 Verona, Italy; federica.vurro@univr.it

**Keywords:** iron oxide nanoparticles, immunotherapy, magnetic resonance imaging, magnetic particle imaging

## Abstract

Starting from the mid-1990s, several iron oxide nanoparticles (NPs) were developed as MRI contrast agents. Since their sizes fall in the tenths of a nanometer range, after i.v. injection these NPs are preferentially captured by the reticuloendothelial system of the liver. They have therefore been proposed as liver-specific contrast agents. Even though their unfavorable cost/benefit ratio has led to their withdrawal from the market, innovative applications have recently prompted a renewal of interest in these NPs. One important and innovative application is as diagnostic agents in cancer immunotherapy, thanks to their ability to track tumor-associated macrophages (TAMs) in vivo. It is worth noting that iron oxide NPs may also have a therapeutic role, given their ability to alter macrophage polarization. This review is devoted to the most recent advances in applications of iron oxide NPs in tumor diagnosis and therapy. The intrinsic therapeutic effect of these NPs on tumor growth, their capability to alter macrophage polarization and their diagnostic potential are examined. Innovative strategies for NP-based drug delivery in tumors (e.g., magnetic resonance targeting) will also be described. Finally, the review looks at their role as tracers for innovative, and very promising, imaging techniques (magnetic particle imaging-MPI).

## 1. Introduction

Imaging biomarkers are gaining increasing importance in cancer studies, due to their ability to provide anatomical (mainly dimensional) and functional characteristics of solid tumors and their microenvironment. Features assessable with PET, CT and MRI include metabolism, the diffusion of tissue water, perfusion, chemical composition and hypoxia. MRI has the unique ability to provide both anatomical and functional information (physiological and pathophysiological) in a single exam. Non-invasive imaging probes such as nanoparticles (NPs) have enormous potential in this area of research: by exploiting the physical and chemical properties of their components (or moieties), they can be made to carry and release anticancer treatments into the target tissue [1,2,3,4], while at the same time acting as diagnostic agents.

In clinical trials, imaging biomarker-based response criteria should help to guide early decisions and minimize patient exposure to unnecessary therapy. Size-based response assessment is generally insensitive to early biological changes, and often fails to identify responses in patients experiencing either cytostasis or pseudoprogression. These conditions are often encountered with new target therapies, where the variability in tumor response increases in comparison to treatment with cytotoxic agents.

Biological changes that occur soon after starting treatment (up to 12 weeks) are apoptosis, necrosis, cystic degeneration, intralesional bleeding, edema and immune cell infiltration. All of these effects are MRI-detectable and can potentially give early evidence of the response to therapy, but anatomical imaging may not identify them, thus compromising clinical decisions. There is therefore a great need for specific MRI biomarkers for new therapies. 

This review will focus on one of the most promising applications of NPs in tumor imaging; specifically, their contribution to the early assessment of the efficacy of immunotherapy, and their capability to alter macrophage polarization.

Immunotherapy is an innovative therapeutic approach based on the stimulation of immune response in tumors. Early response to immunotherapy is represented by recruitment of immune cells at the tumor site, possibly accompanied by reduction in tumor size. The potential of NPs to act as diagnostic agents is correlated to their well-known ability to be internalized by inflammatory cells in vivo [5]. During the last 20 years, their ability to be internalized by different kind of cells, in vitro and in vivo, has been exploited in a number of applications. 

The first section of this review will briefly describe how the ability of iron oxide NPs to enter cells (including stem cells) can provide information on the fate of the cells concerned when transplanted into living organisms, and enable MRI detection of inflammatory cell recruitment. The following sections will focus specifically on applications in cancer immunotherapies. The aptitude of iron oxide NPs to be guided towards lesions will be described in the last-but-one section of the review, while the final section will be devoted to magnetic particle imaging (MPI), an innovative tomographic imaging technology which relies on iron oxide NPs as tracers. Thanks to its high sensitivity, MPI is expected to become an important diagnostic tool in cancer immunotherapy. 

## 2. Iron Oxide Nanoparticles as Contrast Agents for Cellular Imaging

Starting from the mid-1990s, several iron-based MR contrast agents were developed for MRI. They were mainly composed of small (30–200 nm) clusters of iron-containing crystals, forming single magnetic domains and known as ferrites, magnetites, ferumoxides or superparamagnetic iron oxides (SPIOs). In contrast with Gd chelates, iron-based MR contrast agents are characterized by high transverse relaxivity and a high r2/r1 ratio: they are known as T2-relaxing contrast agents. Since they induce strong static magnetic field inhomogeneity in their neighborhood, they can also substantially affect the T2 * relaxation time. Consequently, iron oxide NPs produce a signal loss in a large area surrounding the iron (a so-called “blooming effect”) on T2 *-weighted images [6]. Due to their size, after i.v. injection iron-oxide NPs are preferentially captured by the reticuloendothelial system of the liver and have therefore been proposed as liver-specific contrast agents [7].

**Figure 1:**
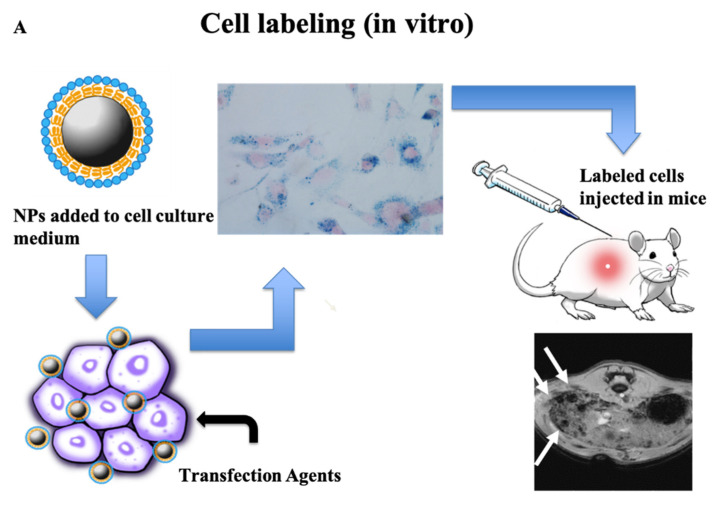

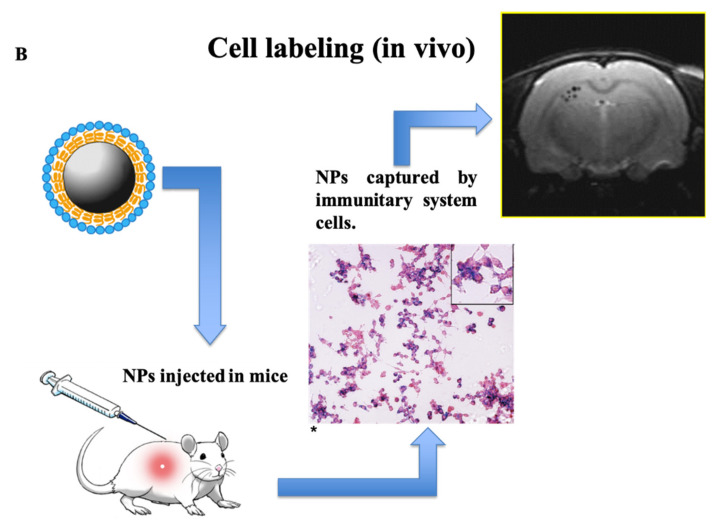
In vitro and in vivo labeling of cells by using iron oxide NPs. (**A**) Experimental protocol adopted to label cells in vitro: NPs are added to the cell culture medium, if possible in the presence of transfection agents. After tests on cell viability and iron content, labeled cells are transplanted into living organisms and their in vivo homing can be visualized by MRI. (**B**) The experimental protocol for in vivo labeling of cells consists in injection of NPs into the bloodstream, where they are captured by immune system cells. MRI therefore allows visualization of regions of immune cell accumulation. The histological image in Figure 1 B(*) is modified from ref. [24].

Thanks to their ready internalization by different kinds of cells, during the last 20 years iron oxide NPs have been widely applied to label and track cells administered as therapy for different pathologies. Figure 1A shows a general overview of the experimental protocol applied. Briefly, NPs are added to the cell culture medium, possibly in the presence of transfection agents. The best experimental conditions, such as time of incubation, concentration of iron-oxide NPs and addition of transfection agents, are determined in terms of cellular iron content and cell viability. Cells are then injected into the recipient body and MRI is finally applied to track the cells in vivo [8]. A number of preclinical studies have used this method to examine the fate of different kinds of cells—e.g., stem cells [9,10,11], pancreatic islets [12,13], dendritic cells [14] or even exosomes extracted from stem cells [15,16].

Preclinical studies have demonstrated advantages and disadvantages of the method. Advantages are its high sensitivity, even reaching the single cell [17,18] and the superb anatomical detail of MRI, allowing elucidation of cell homing and translatability to the clinical setting [19]. The major drawbacks are that it is not possible to distinguish between dead and live cells, that signal void in MRI does not quantitatively report on the number of cells, that the label undergoes dilution with cell replication in vivo and that iron oxide NPs previously approved for clinical use as MRI contrast agents have been withdrawn from the market. An updated list of iron oxide (IO)-based contrast agents that have been clinically approved or entered clinical trials as MRI contrast agents can be found in [20], together with their intended use and status on the market.

In addition, cell internalization of SPIOs can occur in vivo, as reported in Figure 1B. Iron oxide NPs injected into the bloodstream are phagocytosed by circulating monocytes that can migrate into tumors and differentiate into macrophages. MRI can therefore detect in vivo recruitment of immune system cells in tumors, as well as in other organs and tissues. Kirschbaum et al. [21] have recently applied this method to map inflammatory infiltrates, using high-field MRI and iron oxide NPs for cell tracking in an experimental model of multiple sclerosis. They demonstrated that NP uptake is specific for innate immune cells and correlates with clinical severity of the disease. Their results pave the way for clinical translation to a wide variety of inflammatory conditions, for improved diagnosis and treatment monitoring [21]. The same method has been tested in experimental models of organ transplantation, where the recruitment of macrophages is one hallmark of transplant rejection (see, for example, [22]). Studies have also been carried out in clinical settings. In a recent clinical study, macrophage inflammation and myocardial edema were successfully imaged in patients following myocardial infarct, using Ultrasmall SPIO-enhanced T2 * MRI and T2 mapping. Overall, the study demonstrates that the technique can provide a non-invasive method for the diagnosis and monitoring of tissue inflammatory macrophage activity in the heart [23].

Iron oxide NPs have been widely used to identify macrophage content in solid tumors. As mentioned before, this is because iron oxide NPs are predominantly captured by phagocytic cells such as macrophages and Kupffer cells; this means that they do not need to be conjugated with antibodies, enabling their direct intravenous administration and detection by means of a conventional 1 H radiofrequency coil with a T2 *-weighted sequence. To facilitate the detection of spatial distribution of tumor associated macrophages (TAMs), and to allow the quantification of deposited iron, it is possible to collect a T2 * map with a multi-gradient echo sequence; an alternative is to quantify the change in susceptibility caused by the contrast agent via quantitative susceptibility mapping. Both these methods are linearly related to the concentration of iron oxide NPs [25,26]. This review will be mainly focused on studies of tumor immunotherapy, which we see as a relevant application in the near future.

## 3. Immunotherapies and the Role of NPs in Targeting Macrophages

Tumors avoid immune elimination by activating an immunosuppressive microenvironment. Malignant transformation induced by alterations of oncogenes and tumor suppressor genes can lead to the recruitment of an immune response that suppresses antitumor immunity.

TAMs are an important component of the tumor microenvironment. There are two subpopulations of TAMs, with two distinct phenotypes: M1 (proinflammatory and antitumoral phenotype) and M2 (protumoral and anti-inflammatory phenotype). An increased number of TAMs with an M2-like phenotype are associated with poor prognosis, and promote tumor angiogenesis and immune suppression. The new frontiers of immunotherapy include promising strategies to induce the switching of the tumor-promoting immunosuppressive microenvironment (M2)—characteristic of tumors rich in macrophages—to one that kills tumor cells, is anti-angiogenic and promotes adaptive immune responses (M1).

As previously mentioned, starting from the mid-1990s, several iron-based MR contrast agents were developed for MRI. One of the first applications of superparamagnetic iron oxides (SPIOs), in imaging of tumor-associated macrophages, showed that MRI could longitudinally monitor the presence of TAMs during tumorigenesis and that SPIO TAM imaging could thus provide valuable prognostic information. After a single SPIO administration, Shih and co-workers showed that the TAM-associated MR hypointense area serves as an anchor point for tumor expansion, and SPIO-labelled TAMs gradually appeared closer to vessels within 14 days [27]. The authors explain this by hypothesizing that abundant oxygen and nutrients (i.e., an angiogenic switch) are indispensable to achieve both tumor growth and the required vascularization. TAMs are known to induce endothelial cell migration and proliferation by releasing proangiogenic factors. The authors assume that the location of TAMs in the avascular regions of the early tumor is associated with the angiogenic switch, and can produce new vascular networks for tumor growth. Hemorrhagic areas (due to a disorganized, leaky vascular bed) tend to become hypoxic, recruit more macrophages to remove dead cells and release proangiogenic factors. With the progression of tumor growth, TAMs are believed to differentiate into endothelial-like cells and become part of the tumor vasculature. 

The currently most promising contrast agent in TAM-related MRI is ferumoxytol, an FDA-approved, ultra-small iron oxide NP (USPIO) used to treat anemia. It has replaced all previously approved contrast agents for the reticuloendothelial system since manufacturers ceased their commercial production in 2009. Ferumoxytol is a colloidal solution of USPIO NPs, composed of an iron oxide core and a semisynthetic carbohydrate coating of polyglucose sorbitol carboxymethyl ether, with a mean hydrodynamic diameter of 30 nm [28]. Currently used as an alternative contrast agent in patients with impaired renal function, it is being investigated in a clinical trial of glioblastoma treated with immune checkpoint modulators [29]. Its major advantage compared to other contrast agents is its prolonged intravascular residence time (>12 h), thanks to its size and carbohydrate coating.

The ability of ferumoxytol to label TAMs in vivo was first documented by Daldrup et al. [30], in experimental breast cancers in mice. The authors observed initial tumor perfusion, followed by tumor retention and persistent MR enhancement 24 h after intravenous administration. In vitro analyses confirmed that iron oxide NPs were preferentially phagocytosed by TAMs, but not by malignant tumor cells. 

**Figure 2:**
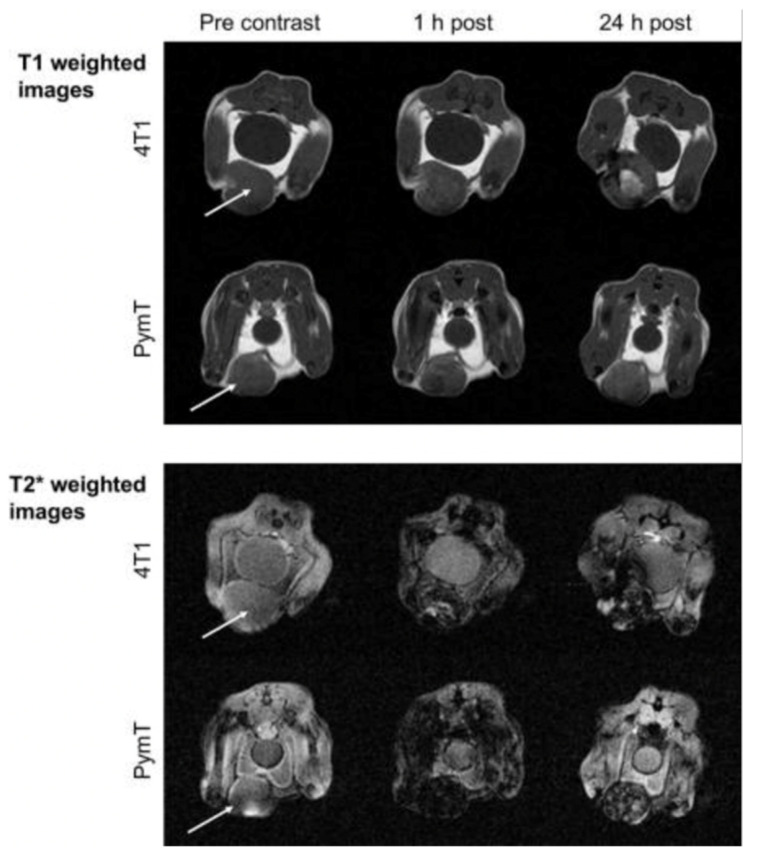
Early necrosis was detected by ferumoxytol in MRI of a fast-growing, aggressive 4T1 mammary tumor and compared with a slow-growing mammary tumor (the MMTV-PymT model). T1- and T2 *-weighted MRI of two representative tumors, pre-injection, 1 h and 24 h post-injection of ferumoxytol (0.5 mmol Fe/kg), showed that both tumors were homogeneous in pre-contrast images, while at 24 h 4T1 tumors exhibited a marked central T1 enhancement. Figures reproduced from ref. [31].

Daldrup and co-workers also showed that differential T1 and T2 enhancement patterns of USPIO in tumors enable conclusions about their intracellular and extracellular location [31]. The authors compared the enhancement pattern in a transgenic MMTV-PymT mouse model (developing spontaneous, slow-growing multifocal cancers of the mammary glands) to a more aggressive, fast-growing malignancy with early central necrosis, resulting from the implant of 4T1 cells in BALB/c mice. They found T1 enhancement, together with darkening in T2 images of histologically confirmed areas of necrosis (free iron oxide nanoparticles), in the 4T1 models. Regions of intracellularly compartmentalized ferumoxytol NPs (such as those present in MMTV-PymT tumors) showed predominant T2, but little or no T1, enhancement (Figure 2). Ferumoxytol imaging in a pilot study on patients showed similar findings [24].

An intrinsic therapeutic effect of ferumoxytol on the growth of early mammary cancers, and lung cancer metastases in liver and lungs, was also shown by Zanganeh and co-workers [32]. Breast tumor cells co-injected with ferumoxytol exhibited a markedly delayed growth rate, as compared to the same tumor cells injected without the addition of ferumoxytol. In addition, systemic delivery of ferumoxytol resulted in a significant reduction of lung and liver metastasis in mice.

The authors isolated macrophages from ferumoxytol-exposed cancer cells and macrophage co-cultures, demonstrating by quantitative real-time polymerase chain reaction (RT-PCR) that ferumoxytol-exposed macrophages upregulated M1-related TNFα and CD86 markers. Moreover, mRNA levels of M2-related CD206 and interleukin-10 markers significantly decreased after exposure to ferumoxytol. Similarly, an enzyme-linked immunosorbent assay (ELISA) of co-cultures showed significantly increased production of tumor necrosis factor-α (TNFα), a classical M1 marker, but no significant production of M2-related interleukin-10. The authors demonstrated that ferumoxytol NPs inhibited cancer growth by inducing a proinflammatory immune response coupled to M1 macrophage polarization. 

Yang and co-workers have shown that innate immune stimulation can be tracked in an animal model of glioblastoma, using ferumoxytol [33]. They started from the hypothesis that glioblastoma-initiating cells (GICs), a subpopulation of quiescent cells, polarize blood-borne monocytes into TAMs with an anti-inflammatory phenotype to promote tumor growth. The authors propose a treatment with the immune stimulator amphotericin B (Amp B), which should reprogram compromised monocytes/macrophages into a pro-inflammatory phenotype that infiltrates the brain and suppresses the tumor. A reduction in T2 *, together with histology, confirmed that Amp B enhanced the transfer of iron into the brain tumor. This was likely caused by the iron that monocytes carried into the tumor (after phagocytosis), since Amp B is known to increase monocyte activation and infiltration in intracranial GBM in mice.

**Figure 3:**
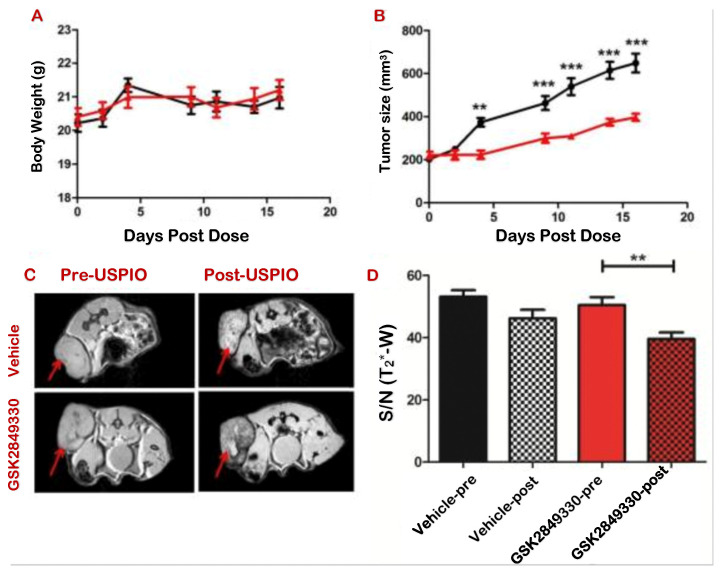
GSK2849330 (an innovative anti-HER3 humanized monoclonal antibody) inhibited the growth of CHL-1 human melanoma by increasing macrophage recruitment, as shown by ferumoxytol-enhanced MRI. (**A**) Maintaining the same body weight, the growth of CHL-1 human melanoma was significantly inhibited in the GSK2849330 treated group (red) as compared with the vehicle group (black), ** *p* < 0.01, *** *p* < 0.001 using 2 way ANOVA (**B**). (**C**) T2 *-w MRI pre- and 24 h post-ferumoxytol injection. Red arrows indicate tumor location. (**D**) Tumor signal/noise ratio (S/N (T2 *-w)) significantly decreased post-ferumoxytol injection in the GSK2849330 treated group. ** *p* < 0.01: Unpaired *t*-test (two-tailed). Figure reproduced from ref. [34].

Ferumoxytol-enhanced MRI was also used to monitor aspects of the immune system response to an innovative anti-HER3 humanized monoclonal antibody [34]. ErbB3/HER3 is a member of the epidermal growth factor receptor family of receptor tyrosine kinases, which play an important role in the development and progression of cancer. The authors studied the distribution of this innovative anti-HER3 humanized monoclonal antibody (GSK2849330) in vivo by using a variety of imaging techniques, including ferumoxytol -enhanced MRI. They directly tracked the antibody with PET and optical imaging, while ferumoxytol-enhanced MRI showed the macrophage recruitment—i.e., the biological marker of in vivo response in preclinical models. The authors found a decreased signal-to-noise ratio 24 h post CA administration in the treated groups, indicating that USPIO uptake was higher in the GSK2849330-treated group than in the vehicle group, and was associated with tumor growth reduction (Figure 3). The significant increase in tumor macrophages was confirmed by a quantitative immunohistochemistry analysis. Combined use of PET and MRI will potentially result in clinically translatable, non-invasive techniques to assess immune activation and anti-tumor responses.

**Figure 4:**
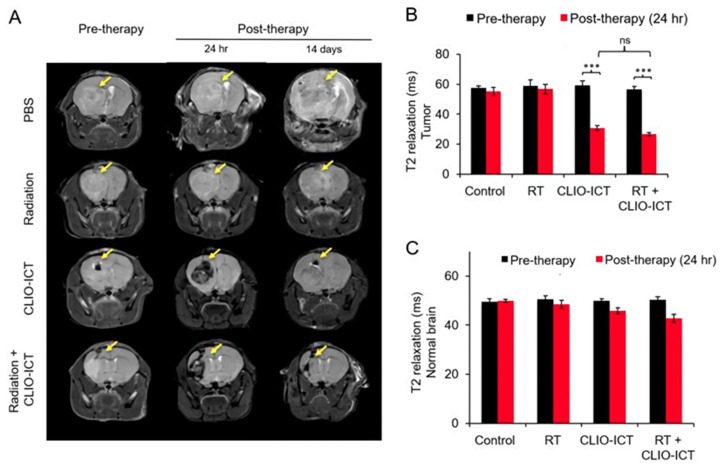
The effect of the vascular disrupting agent azademethylcolchicine (ICT2552) conjugated with the cross-linked iron oxide nanoparticle CLIO-ICT, in combination with radiation therapy, was monitored by MRI. (**A**) Representative coronal T2-weighted MR images of the brain of NSG™ mice with GBM39 glioblastoma tumors, before and after CLIO-ICT (10 mg/kg ICT, 34 mg/kg Fe) and/or radiation (10 Gy) treatment (as monitored at 24 h and on 14 days). Hypointense tumor enhancement in animals treated with CLIO-ICT and CLIO-ICT + radiation confirms theranostic nanoparticle delivery. Corresponding T2 relaxation times of tumors (**B**) and normal brain (**C**), before and 24 h after therapy. *** *p* < 0.001, ns = not significant. Figure reproduced from ref. [35].

A cross-linked iron oxide NP (CLIO) conjugated with a highly effective vascular disrupting agent, azademethylcolchicine (ICT2552), has been recently developed to target and eradicate the subpopulation of GIC cells, which could be a reason for radioresistance and relapse in glioblastoma patients [35]. Glioblastoma (GBM) is the most common and the most aggressive primary brain tumor. Even though innovative therapeutic strategies have been developed, patients often relapse. The standard treatment includes resection and post-operative radiotherapy. GICs are a subpopulation of quiescent cells that can escape the toxic effect of radiation by activating DNA damage repair pathways. Increasing doses of radiation are not suitable to target GICs, because they will lead to systemic toxicity. More effective strategies for GIC targeting should include the combination of radiation with another treatment, such as a vascular disrupting agent. The authors found that the theranostic NP CLIO-ICT combined with radiation therapy significantly reduced microvessel density and the number of GICs, increased caspase-3 expression and prolonged the survival of glioblastoma-bearing mice. In addition, MRI (Figure 4) effectively monitored NP delivery to the tumor.

## 4. The Role of NPs in Oncolytic Virotherapy

Oncolytic virotherapy consists in the lysis of tumor cells, mediated by viral infection of tumors. Several virus strains are able to destroy tumor cells selectively. In addition to this primary effect, a massive inflammation within the tumor microenvironment occurs. The recruitment of inflammatory cells in the tumor is primarily directed against the virus. However, virus-mediated cell lysis also leads to the release of tumor-associated antigens, which in turn stimulate an anti-tumor immune response, such as the recruitment of macrophages and T lymphocytes, as a secondary effect. The latter produce cytokines that can recruit other immune cells and actively destroy cancer cells. Both innate and adaptive immune responses generate an immunological memory that prevents cancer recurrence and synergizes with the oncolytic action of the viruses. 

Several studies have assessed the efficacy of oncolytic virotherapy in preclinical and clinical studies, mainly in melanoma and brain tumors. MRI can monitor the intratumoral inflammation that is produced during oncolytic virotherapy. By using 19F MRI [34] and iron oxide NPs [35], it is possible to indirectly detect and quantify virus localization, monitor the therapeutic outcome of oncolytic virotherapy and optimize new therapeutic virus strains.

**Figure 5:**
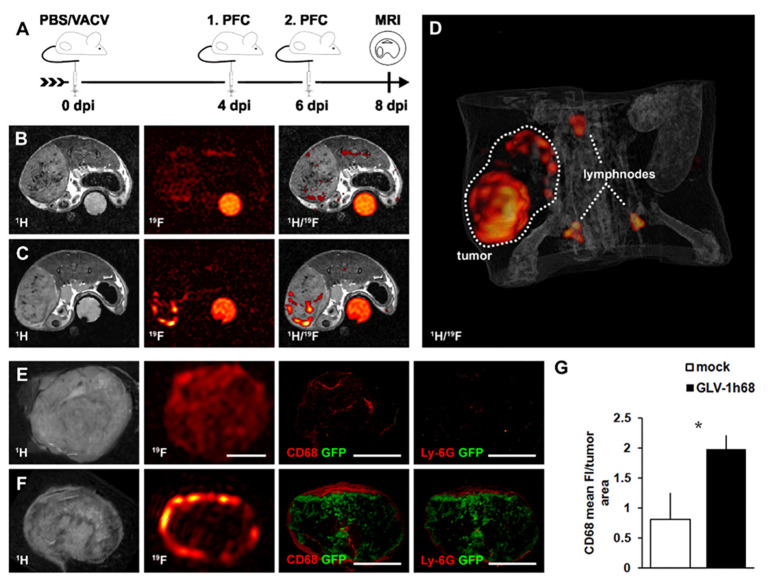
Visualization of viral colonization within tumors by 19F MRI. (**A**) Timeline-1936-MEL melanoma-bearing, athymic nude mice were injected i.v. with virus (1 × 107 pfu of GLV-1h68) or PBS as a control. Emulsified PFC was administered i.v. on days 4 and 6 post virus injection, followed by 19F MRI 48 h later. (**B**,**C**) Representative 1H, 19F and overlaid 1H/19F in vivo images of a mock-infected, tumor-bearing mouse (**B**), showing a low 19F signal throughout the tumor tissue; and a GLV-1h68-infected, tumor-bearing mouse (**C**), exhibiting intense 19F signal accumulation along the tumor rim. (**D**) 3D 1H/19F overlay reconstruction of the abdomen of a GLV-1h68-infected mouse clearly showed the accumulation of PFC in the tumor and adjacent lymph nodes. (**E**,**F**) Representative ex vivo 1H, 19F images together with corresponding histology (CD68, Ly-6G), showing the distribution of the CD68+-macrophage/Ly-6G+-neutrophil population and the 19F signal of a mock-infected tumor (**E**) and a GLV-1h68-infected tumor (**F**). (**G**) Quantitative analysis of CD68-fluorescence intensity (FI) of GLV-1h68-colonized tumors show a marked increase over mock-infected control tumors. * *p* < 0.05; Students *t* test. Figure reproduced from ref. [36].

Weibel and co-workers [36] provided a longitudinal, non-invasive quantification of intratumoral inflammation during oncolytic virotherapy, using perfluorocarbon nanoemulsions (PFC) and 19F MRI. By comparing in vivo and ex vivo 19F/1H MRI with histology, the authors demonstrated that tumor viral colonization significantly altered 19F signal distribution and intensity in solid tumors as well as in the adjacent lymph nodes, indicating the potential of this imaging modality for the localization of the host immune response and for sentinel lymph node detection (Figure 5). The comparison revealed a widespread distribution of both the 19F signal and the CD68+-macrophages throughout the mock-infected tumors, while viral-infected tumors showed 19F-positive hot spots only along the tumor rim. A similar distribution pattern was observed for the CD68+-macrophage population. These results indicate that, rather than labeling intratumoral TAMs, PFC NPs are likely to label the circulating immune cells that immigrate into the tumor after viral colonization.

The feasibility of detecting and quantifying PFC NPs corresponding to areas of virally induced recruitment of macrophages in vivo, using 19F MRI, highlights its potential for therapeutic monitoring and as a prognostic indicator of therapeutic outcome.

## 5. NPs for Magnetic Resonance Targeting (MRT)

Recently proposed, innovative therapeutic approaches exploit the magnetic properties of SPIO-labelled NPs and an external magnetic field or magnetic field gradient coils (present in all MRI systems), to steer ferromagnetic particles (or cells containing them) to a target site (primary tumor or metastasis) [37]. These methods are particularly attractive for all cell-based therapies that have to deliver a therapeutic agent (such as a protein or virus), or bone marrow-derived cells (such as T cells, dendritic cells, macrophages and stem cells). More often than not, only a small proportion of these cells reach the tumor site; increased precision of targeting would improve their therapeutic efficacy and reduce the risk of side effects. 

In solid tumors, macrophages were found to accumulate in poorly vascularized areas in mice and humans. Muthana and co-workers hypothesized that these cells could be used to deliver therapeutic agents to these poorly vascularized—and thus relatively inaccessible—areas of tumor. MRT was used to direct macrophages carrying an oncolytic virus from the bloodstream into primary and metastatic tumor sites in mice [37].

The authors first demonstrated that actuation forces could be generated by a preclinical 7 T MRI system, using pulsed gradients of 300 mT/m amplitude with an effective additional magnetic field offset (B_0_) of about 1.5 mT at the target site. The authors steered human SPIO-loaded macrophages across a trans-endothelial migration flow chamber (consisting in a perforated membrane coated with a layer of human vascular endothelial cells, thereby mimicking flow in tumor venules), into three-dimensional human multi-cellular tumor spheroids cultured below the membrane. The authors found a T2 *-weighted signal loss (indicating higher concentration of iron) in spheroids after MRT, compared with the control samples (where gradients were not pulsed).

They then detected significantly increased in vivo uptake of SPIO-loaded macrophages in primary prostate tumors in mice after MRT, without alteration of the function or integrity of the tumor vasculature, vascular perfusion and permeability (determined by in vivo dynamic contrast-enhanced MRI and ex vivo staining). 

Finally, the authors assessed the therapeutic benefits of MRT by administering macrophages loaded with SPIO NPs and an oncolytic virus (HSV1716) to tumor-bearing mice. MRT increased the antitumoral effects of oncolytic macrophages: the growth of primary tumors was reduced, the percentage of necrosis increased and regrowth was delayed.

These results have opened up the possibility of real-time, image-guided targeting with an MRI system to improve treatment efficacy and reduce side effects, but optimization for targeting as well as physiological and hardware constraints have so far limited its development and application.

A more recent study focused on the ability of magnetic NP-loaded T cells to be targeted and retained in vitro and in vivo, at a site of interest, with an external magnetic field (EMF) [38]. In this respect, one of the main limitations of cell-based therapies is the dispersion of the systemically administered cells in vivo. New strategies that promote specific cell infiltration and accumulation in target tissues have been developed and tested for non-lymphoid cells (stem cells, mesenchymal cells, macrophages or dendritic cells) loaded with NPs. However, there are still challenges regarding in vivo delivery of magnetically guided, highly motile effector lymphoid cells, such as T or natural killer cells, with a view to targeting, and accumulating them in, specific regions such as lymph nodes (LNs) or solid tumors. During an immune response, T cells interact with other cells and detect antigens in different tissues. T cell migration is regulated by multiple factors and by the interaction with other cells in secondary lymphoid organs such as the lymph nodes, where tissue organization allows primary T cells to encounter the antigen-presenting cells coming from different tissues and become activated. Once activated, these antigen-experienced T cells scan peripheral tissues to find and eliminate their antigen. To improve the retention of activated T cells in a target tissue could be a promising strategy to enhance the immune response.

**Figure 6:**
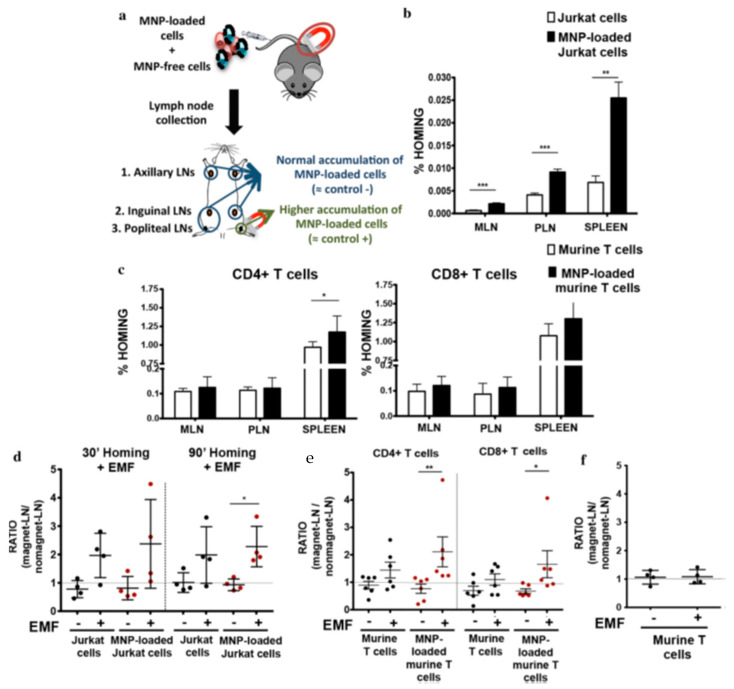
Electromagnetic field (EMF) capacity of steering in vivo Jurkat and murine primary T cells towards lymph nodes, after treatment with magnetic nanoparticles (MNP). (**a**) Experimental set-up for determining the homing capacity of MNP-loaded cells as compared with MNP-free cells. A mixture of MNP-free and MNP-loaded human T cells (Jurkat) or murine T cells was prepared and injected i.v. into nude or C57BL/6J mice, respectively. Homing capacity of Jurkat (**b**) and murine T cells (**c**), loaded or not with MNP in the absence of an EMF, as measured by flow cytometry. Student’s *t*-test, * *p* < 0.05, ** *p* < 0.01, *** *p* < 0.001. Ratio of MNP-free and MNP-loaded Jurkat (**d**) and murine (**e**) T cells, in the EMF-exposed lymph node (LN) to the control LN (no EMF), 20 min after i.v. injection of the cell mixture in recipient mice. (**f**) Ratio of MNP-free murine T cells, administered i.v. as a control, in the EMF-exposed LN to control LN (no EMF). Student’s *t*-test, * *p* < 0.05, ** *p* < 0.01, *** *p* < 0.001. Figure reproduced from ref. [38].

The authors synthesized and characterized magnetic NPs with coatings that provide different surface charges (dimercaptosuccinic acid (DMSA), 3-aminopropyl-triethoxysilane (APS) or dextran) and studied their effect on T cells. As expected because of their reduced cytoplasm, T cells were unable to internalize the NPs, which remained on the cell surface (in close contact with cell membrane). After adoptive transfer using in vivo models, NP-loaded T cells were retained to a greater extent in lymph nodes exposed to an EMF, which was shown to favor their retention (Figure 6). The combined use of magnetic NPs and EMFs did not alter T cell viability or function, and promoted the retention of T cells in vivo, which could be implemented in adoptive cell transfer therapies in mice. 

The last example of magnetic targeting by an EMF exploits ferroptosis, another intrinsic effect of magnetic iron oxide NPs on tumors. Ferroptosis is a newly proposed, programmed cell death mechanism, characterized by mitochondrial lipid peroxidation and reduced levels of glutathione peroxidases, which leads to the accumulation of lipid peroxides [39,40]. At low pH, iron ionization together with a high concentration of H_2_O_2_ species can induce a localized Fenton reaction, leading to the generation of ·OH species that exert a damaging action on tumor cells. The authors demonstrated that amorphous iron NPs can be used as theranostic agents, benefiting from their glassy nature [41]. In this respect, amorphous iron NPs showed several advantages over the corresponding nanocrystalline particles, because amorphization (disordered atomic arrangement) may improve their biodegradability and magnetism-related properties (for magnetic targeting). The authors demonstrate that intratumoral amorphous iron NP administration inhibited the growth of 4T1 mammary carcinoma implants in mice. In addition, systemic (i.v.) delivery of the amorphous iron NPs showed a limited effect on tumor volume reduction. This effect was more evident in the presence of an external magnetic field, due to the magnetic targeting of the NPs on tumors. 

## 6. Recent Advances in Imaging Techniques: Magnetic Particle Imaging

Magnetic particle imaging (MPI), an innovative imaging technique that is specific, quantitative and sensitive for the detection of iron oxide NPs, has emerged recently. MPI makes it possible to overcome some MRI limitations, including low specificity (owing to other low signal regions in MRI, such as hemorrhagic regions or those containing air) and difficult quantification. MPI directly detects iron oxide NPs, which produce positive contrast with no underlying background signal from biological tissues, resulting in high tracer specificity. Detection limits as low as 1.1 ng iron (Fe), and a concentration of 550 pg Fe/μL in vitro [42] and 7.8 ng Fe in vivo [43], have been demonstrated. In MPI, the signal is detected using a static gradient field with a single field-free region (FFR), which can be a point or a line. An alternating magnetic field is then applied to generate signal from particles present in the FFR. Images are generated by raster scanning the FFR across the entire field of view. Superparamagnetic particles outside the FFR remain saturated in magnetization, and do not contribute to the signal [44].

MPI directly detects SPIO magnetization, as a result of which the signal is highly dependent on the physical characteristics of the SPIO tracer [45]. To be suitable for MPI, NPs should have three characteristics: they should be superparamagnetic, receptive to magnetic saturation and exhibit a non-linear magnetic curve [46].

Many SPION agents for MRI possess the above-mentioned characteristics and have therefore been evaluated for their possible application in MPI. Resovist^®^, one of the previously developed MRI contrast agents for the liver [20], became the gold standard for MPI. Recently, Magnetic Insight, Inc. has commercialized a carboxydextran-coated iron oxide NP formulation as VivoTrax^®^, with the same reference standard. Interestingly, the clinically approved ferumoxytol can be readily detected with MPI [47]. Since neither Resovist^®^ nor VivoTrax^®^ were shown to be ideal MPI candidates, many research groups are active in the synthesis of new MPI tracers.

MPI is currently being explored in several biomedical applications. The high sensitivity of MPI is suitable for early tumor detection [48], where MPI takes advantage of the enhanced permeability and retention (EPR) effect in tumors. The EPR effect is caused by leaky vessels with large pores, making tumor tissue ideal for nanomedicines and nanosized contrast agents. MPI therefore has great clinical application value in the early diagnosis of cancer.

Cell tracking is among the earliest and more promising applications of MPI, thanks to its high tissue penetration, the absence of background noise and a high degree of sensitivity that enables it to detect as few as 200 labeled cells [48]. Nejadnik et al. have recently applied MPI to in vitro and in vivo detection of mesenchymal stem cells (MSCs) labeled with clinically translatable feromuxytol NPs [45]. MPI allowed accurate in vivo detection and quantification of ferumoxytol-labeled stem cells, an important finding for clinical translation of MPI technologies.

Zheng et al. [49] used MPI to detect the dynamic trafficking of i.v. MSC administration. MSCs were labeled using Resovist^®^, and the results indicated that labeled MSCs are immediately entrapped in lung tissue and then clear to the liver within one day. Overall, this paper showed the use of MPI to dynamically track the systemic administration of human mesenchymal stem cells.

Wang et al. [50] first reported the application of MPI to monitoring transplanted islets in the liver and under the kidney capsule in experimental animals. Pancreatic islets were labeled using VivoTrax (Magnetic Insight Inc., Alameda, CA, USA). Imaging of labeled islet phantoms revealed a direct correlation between the iron content obtained from MPI image analysis and the number of agarose-embedded islets. In vivo, labeled pancreatic islets were detectable both in the liver and under the kidney capsule. MRI and MPI results were consistent.

MPI is currently being applied in the detection of TAM as well. Makela et al. [43] recently applied MPI to in vivo tracking of TAMs in comparison with MRI. Briefly, ferumoxytol or Vivotrax were injected i.v. into mice bearing experimental breast tumors (4T1 cells), The mice were imaged by using both MRI and MPI, in vivo and ex vivo. The results demonstrated that ferumoxytol was superior to Vivotrax for TAM imaging by MPI, and that MPI provides quantitative information on in vivo iron labeling of macrophages that is not attainable by MRI. Inter alia, MPI provided information in organs such as the lungs, where MRI loses its diagnostic usefulness.

It is worth mentioning that SPIONs can generate MPI signals and heat simultaneously: by using these particles, MPI can become a theranostic platform for precise, real-time visualization of magnetic hyperthermia [51]. According to the MPI imaging principle, only the particles within the FFR respond to the high-frequency alternating magnetic field (AMF) and generate energy to heat surrounding tissue. In contrast, particles covered by the gradient field but outside the FFR will not generate heat, even within the AMF. As a result, if the frequency of the AMF is intense enough, the magnetic NPs within the FFR will be excited by the field and generate adequate thermal energy, thereby causing cancer cell death.

To date, MPI has led to significant progress in different areas, such as cell tracking, tumor imaging, drug delivery, blood pool imaging and magnetic hyperthermia visualization. More applications of MPI in the fields of diagnosis and treatment should be explored, taking advantage of such characteristics as high resolution, high sensitivity, unlimited penetration depth, linear quantitation and real-time imaging.

## 7. Conclusions

As innovative cancer immunotherapies become increasingly relevant in the fight against cancer and are translated from preclinical investigations to clinical practice, the need for non-invasive imaging techniques that can quantify macrophage responses increases accordingly. Even though several imaging methods are available, including PET, Gd-enhanced MRI [52] and 19F MRI, the application of MRI with superparamagnetic iron oxide contrast agents is probably the most promising. These NPs offer major advantages, including detectability by MRI, theranostic properties, deliverability to the target by magnetic gradient actuation forces and multimodal imaging capability (MRI-MPI). In addition, although clinical development of SPIOs has been discontinued, some contrast agents such as Resovist^®^ or Feromuxytol are currently available on the market.

We must mention that some concerns about the clinical translation of IO-NPs have been raised in relation to their in vivo fate and possible toxicity. When cleared from the bloodstream, IO-NPs are mainly found in the liver and spleen with small evidences in the lungs, kidney, heart and even brain [53]. In the liver, NPs are encapsulated in phagocytic vesicles or phagosomes. The latter fuse with lysosomes and degrade NPs by digestive en-zymes. The main four criteria for safety consideration (absorption, distribution, metabolism and excretion) are strongly dependent on NPs characteristics, such as size, size distribution, surface charge, coating molecules and protein corona formation. The above mentioned paper [53] concludes that published results often show inconsistencies regarding the potential side effects of IO-NPs. Thus more data from future studies involving systematic analysis of well-characterized particles under clearly defined experimental conditions are needed for clinical translation of IO-NPs based methods.
